# Human polyomaviruses and incidence of cutaneous squamous cell carcinoma in the New Hampshire skin cancer study

**DOI:** 10.1002/cam4.674

**Published:** 2016-02-21

**Authors:** Anala Gossai, Tim Waterboer, Anne G. Hoen, Shohreh F. Farzan, Heather H. Nelson, Angelika Michel, Martina Willhauck‐Fleckenstein, Brock C. Christensen, Ann E. Perry, Michael Pawlita, Margaret R. Karagas

**Affiliations:** ^1^ Geisel School of Medicine at DartmouthHanoverNew Hampshire; ^2^ German Cancer Research Center (DKFZ)HeidelbergGermany; ^3^ New York UniversityNew York, New York; ^4^ Masonic Cancer CenterUniversity of MinnesotaMinneapolisMinnesota

**Keywords:** Cutaneous squamous cell carcinoma, epidemiology, polyomavirus, serology, skin cancer

## Abstract

Squamous cell carcinoma (SCC) of the skin is a malignancy arising from epithelial keratinocytes. Experimental and epidemiologic evidence raise the possibility that human polyomaviruses (PyV) may be associated with the occurrence of SCC. To investigate whether the risk for SCC was associated with PyV infection, seropositivity to 10 PyV types was assessed following diagnosis in a population‐based case–control study conducted in the United States. A total of 253 SCC cases and 460 age group and gender‐matched controls were included. Antibody response against each PyV was measured using a multiplex serology‐based glutathione S‐transferase capture assay of recombinantly expressed VP1 capsid proteins. Odds ratios (OR) for SCC associated with seropositivity to each PyV type were estimated using logistic regression, with adjustment for potentially confounding factors. SCC cases were seropositive for a greater number of PyVs than controls (*P *= 0.049). Those who were JC seropositive had increased odds of SCC when compared to those who were JC seronegative (OR = 1.37, 95% CI: 0.98–1.90), with an increasing trend in SCC risk with increasing quartiles of seroreactivity (*P* for trend = 0.04). There were no clear associations between SCC risk and serostatus for other PyV types. This study provides limited evidence that infection with certain PyVs may be related to the occurrence of SCC in the general population of the United States.

## Introduction

Cutaneous squamous cell carcinoma (SCC) is a keratinocyte cancer (KC), with increasing incidence rates reported in the United States 1, 2, 3, 4, Australia 5, 6, and Europe 7, 8. Estimates of up to 420,000 individuals in the United States were diagnosed with incident SCC in 2012, and 8800 SCC patients died from this malignancy 2. Established risk factors include elderly age 1, 4, 9 and ultraviolet light 10, 11, but environmental exposure to ionizing radiation 12, 13, arsenic 14, 15, 16, 17, and polycyclic aromatic hydrocarbons 18, 19 further increases the risk of SCC. Inherited traits such as skin pigmentation 20, 21 and genetic defects in the ability to correct DNA photolesions (e.g., xeroderma pigmentosum) 22, 23, 24 also contribute to risk. While the prognosis is generally favorable, metastases can occur; and the aging population, as well as disfigurement and morbidity resulting from SCC, makes this malignancy an important public health concern and under‐recognized health burden.

Immunocompromised persons (e.g., organ transplant recipients, and those given immunosuppressant medications) carry a higher risk of SCC 1, 4, 25, 26, 27, 28, raising the possibility of a viral etiology. However, the detection of viral sequences in tumors can reflect either a causal or bystander role 29, 30. Thus far, genus *β* human papillomaviruses (HPV) 31, 32 have been associated with an increased risk of SCC, but a causal relationship has not yet been established in the general population 33.

A potential etiologic role for polyomaviruses (PyV) in cancer has been investigated, and a rapidly expanding number of viral types are being identified in the family *Polyomaviridae* (reviewed in DeCaprio & Garcea, 2013 34). PyVs are DNA viruses with an icosahedral capsid ~45 nm in diameter containing a circular double‐stranded genome 35, 36 that encodes capsid proteins (VP1, VP2, and VP3), as well as small and large T antigens (TAg) 35. In simian virus‐40 (SV40), a PyV naturally infecting Asian macaques 37, 38, the large TAg possesses tumorigenic properties, including the ability to bind and thereby inactivate tumor suppressor proteins Rb 39 and p53, 40 stimulating host cell cycle 35.

While the ability of PyVs to cause tumors in vitro and in experimental systems is undisputed, their role in human malignancies—and specifically KCs—is just beginning to emerge. Multiple human PyVs show evidence of skin tropism, including Merkel cell polyomavirus (MCV), *Trichodysplasia spinulosa*–associated polyomavirus (TSV), and human polyomaviruses 6 and 7 (HPyV6 and HPyV7) 36. MCV was initially discovered in Merkel cell carcinoma (MCC) of the skin 41, and integration of the viral genome in MCC tumors 42 with potentially carcinogenic mutations in the large TAg 43 supported a causal role for MCV in MCC development. Recently, a clinic‐based case–control study from Florida, USA, found an increased SCC risk associated with antibodies against MCV measured following diagnosis, and the association was stronger among those with tumors containing MCV viral sequences 44. Conversely, a case–control study conducted within organ transplant recipients found no evidence of an association between SCC development following transplant surgery and the seroprevalence of numerous PyV types prior to transplantation 45. Further, a large prospective Swedish study found no association between SCC and JC seropositivity assessed prior to diagnosis 46.

Limited epidemiologic research exists on the role of PyV infections (apart from MCV) in SCC carcinogenesis. We therefore sought to investigate the potential association between PyVs and SCC by performing a comprehensive serologic analysis of the frequency of antibodies to the first 10 identified human PyVs: BK virus 47; JC virus 48; Karolinska Institute polyomavirus (KI) 49; Washington University polyomavirus (WU) 50; MCV 41; HPyV6 and HPyV7 51; TSV 52; human polyomavirus 9 (HPyV9) 53; and Malawi/human polyomavirus 10 (HPyV10) 54 in plasma samples collected as part of a large, population‐based case–control study conducted in New Hampshire, USA.

## Materials and Methods

### Study population

The New Hampshire Skin Cancer Study population and methods have been described in detail elsewhere (epidemiologic study design described in Karagas et al., 1998 55) 32, 56, 57. Briefly, histologically‐confirmed, incident SCC cases were identified through active surveillance of dermatology and pathology laboratories throughout New Hampshire, USA. We selected all identified SCC cases diagnosed between July 1993 and June 1995, during the first enrollment phase of this large, population‐based case–control study. Controls were selected from lists of New Hampshire residents obtained from the New Hampshire Department of Transportation (<65 years) and Medicare enrollment lists (≥65 years), and frequency‐matched to the age (25–34, 35–44, 45–54, 55–64, 65–69, and 70–74 years) and gender distribution of cases. For the purpose of the interview, controls were randomly assigned a reference date that matched a case's diagnosis date. To be eligible, participants were required to be residents of New Hampshire, aged 25–74 years at time of diagnosis, speak English, and have a listed telephone number. We excluded participants with squamous cell or basal cell carcinomas on genital sites.

Study participants completed an extensive, structured interview, usually in their homes. Personal interviews were conducted to obtain sociodemographic information (e.g., level of education), lifestyle factors (e.g., cigarette smoking), sunlight‐related characteristics (e.g., response to first exposure in summer to 1 h of sunlight, number of severe sunburns, and skin color), and medical history (e.g., prior history of skin cancer, use of oral glucocorticoids for 1 month or longer, and organ transplant status). Data on the primary tumor(s) (e.g., SCC anatomical location) were collected from a medical records review. Tumors diagnosed as a recurrence of a previously treated tumor, and those appearing contiguous with a scar from a previously excised skin cancer, were not considered a new primary SCC and thus were excluded. All participants provided informed consent in accordance with the Committee for the Protection of Human Subjects at Dartmouth College.

### Human polyomavirus serology

As part of the New Hampshire Skin Cancer Study, we requested a blood sample from all participants for use in future research 55. We collected venous blood samples of 20–30 mL in heparinized tubes following SCC diagnosis (as described in 32, 56). Blood was separated by centrifugation at 2500*g* for 20 min at 4°C, and each component (plasma, red blood cells, and buffy coat) was labeled and stored separately at −80°C until analysis. Specimen label did not reveal the case–control status of the study participant. Samples were shipped to the German Cancer Research Center (DKFZ; Heidelberg, Germany) on dry ice for analysis.

Plasma samples were assayed for antibodies against the immunodominant VP1 capsid protein 58 of 10 human PyVs (BK, JC, KI, WU, MCV isolate 344, HPyV6, HPyV7, TSV, HPyV9, and HPyV10). Plasma samples were also tested for antibodies against the TAg of selected PyV types (large TAg for BK, JC, MCV, HPyV6, HPyV7, TSV, HPyV10, and small TAg for MCV). In our prior study, we did not find strong positive correlations or evidence of cross‐reactivity between the VP1 capsid proteins of most PyV types (data not shown), suggesting that risk estimates obtained during analysis would be specific to that PyV type 59. However, the strong positive correlations between TAg seroreactivities from various PyVs suggestive of assay cross‐reactivity (Fig. S1), and the small number of participants TAg seropositive, resulted in the exclusion of TAg serostatus from the presented analyses. The multiplex antibody detection approach was based on a glutathione S‐transferase (GST) capture enzyme‐linked immunosorbent assay (ELISA) method in combination with fluorescent bead technology (Luminex Corp., Austin, Texas) 60, 61. Antigen preparation and techniques used for PyVs 44, 58, 62 closely follow methods applied to HPVs as described previously 60, 63.

Seroreactivity against PyV VP1 proteins was expressed as the median fluorescence intensity (MFI) of 100+ beads of the same internal color 61. MFI values reflect antibody affinity, titer, and reactivity determined by dilution series 64. Standard cut points to define seropositivity were chosen for each PyV by visual inspection of frequency distribution curves (percentile plots) for the inflection points of all sera tested, as done in prior studies 56, 62, 63, 65. The standard cutoff value for VP1 was 250 MFI units for all 10 PyVs (as used in Teras et al., 2014 66 and Gossai et al., 2016 59). To evaluate the robustness of odds ratio (OR) estimates for SCC by PyV seropositivity, we used a sliding cut point between 50 and 450 MFI units, and also calculated cut points from controls using a method adapted from van der Meijden et al., 2013 67 (Fig. S2). Given the stability of ORs to cut point definition, we ultimately used the standard cut points in all analyses.

### Statistical analysis

Individual characteristics of SCC cases and controls were compared using the X^2^ test (for categorical variables, i.e., gender, education, smoking status, skin color, skin sun sensitivity, number of sunburns, prior KC, glucocorticoid use) or Fisher's exact test (for categorical variables with small strata, i.e., transplant recipients), and Wilcoxon rank sum test (for continuous variables, i.e., age, mean number of PyVs seropositive). Among controls, we previously published the seroprevalence of each PyV type, and tested the association between various individual characteristics in relation to PyV seropositivity; therefore, these analyses were not repeated in this study 59.

We used unconditional logistic regression to calculate the ORs and 95% confidence intervals (CI) for SCC by VP1 seropositivity compared to seronegativity for each PyV type, while adjusting for age group and gender (as used in the frequency matching). We also examined seropositivity for multiple PyV types (7–8 and 9–10 types positive compared with 1–6 types positive), and calculated a *P* for trend based on these categories and a continuous variable of the number of types seropositive. Quartiles of seroreactivity based on the control distributions of continuous MFI values were created for each PyV, and associated with SCC by comparing the second, third, and fourth quartile to the first (lowest) quartile. Tests for trend were conducted by including an ordinal variable in the logistic model. In addition, we evaluated seropositivity for multiple cutaneous PyVs (i.e., MCV, HPyV6, HPyV7, and TSV), and whether seropositivity for all 4 or ≤3 cutaneous PyVs, was associated with SCC risk.

Potentially confounding covariates included level of education, smoking status, eye color, hair color, skin color, skin sensitivity to the sun, number of lifetime painful sunburns, lifetime sun exposure, and the self‐reported use of oral glucocorticoids for 1 month or longer. As none of these factors consistently produced a >10% change in OR 68 when individually placed in a logistic regression model including the frequency matching factors (age group and gender), our OR estimates were ultimately adjusted for only these factors.

We assessed the potential modifying effects of prolonged oral glucocorticoid use for reasons other than organ transplantation, as PyVs have been related to skin conditions and cancers in immunosuppressed populations. In these stratified analyses, we classified participants as users if they reported taking glucocorticoids for 1 month or longer, and excluded those who reported having had an organ transplant. An association between SCC risk and seropositivity to multiple cutaneous *β* HPVs has been reported previously 31, 32. Therefore, using published serologic data from the New Hampshire Skin Cancer Study 31, 32 on 16 cutaneous *β* HPVs (specifically, 5, 8, 20, 24, 36 (beta‐1); 9, 15, 17, 23, 38, 107 (beta‐2); 49, 75, 76 (beta‐3); 92 (beta‐4); and 96 (beta‐5)), we stratified participants by those with seropositivity to 0–1 or seropositivity to ≥2 *β* HPV types to assess possible effect modification by concomitant *β* HPV infections. We further calculated separate ORs for SCC occurring exclusively on anatomical sites with chronic sunlight exposure (head or neck) and other body sites in comparison to controls.

As sensitivity analyses, we excluded participants with a history of organ transplantation (*n* = 7), restricted to participants with no previous skin cancers (neither a prior SCC nor BCC; *n* = 423 controls and 179 cases), and excluded participants with a concomitant basal cell carcinoma (*n* = 22 cases) to assess whether the OR estimates differed from those obtained for all participants. No appreciable change in results was detected (Fig. S3), and thus these individuals were included in the presented analyses. All statistical tests were two‐sided and significance was assessed at the *α *= 0.05 level. Statistical analyses were performed in R version 3.0.1.

## Results

### Study population characteristics

Plasma samples for PyV serology were obtained from 253 (86.3%) of the 293 interviewed SCC cases following diagnosis, and 460 (85.2%) of the 540 interviewed controls (excluding 1 control with an insufficient bead count during serologic analysis). No appreciable differences were noted in the characteristics of individuals for whom we did not obtain serology data (data not shown). Compared to controls, SCC cases were older, were of lighter skin color, had skin that tended to burn following sun exposure, reported a greater number of painful sunburns in their lifetime, were more likely to have had a prior KC (SCC and/or BCC), and were more likely to have had an organ transplant (Table 1).

**Table 1:** Selected characteristics of cutaneous squamous cell carcinoma (SCC) cases and controls from the New Hampshire Skin Cancer Study (*n* = 713).^a^

Variable	SCC cases (*n *= 253),No. (%)	Controls (*n *= 460),No. (%)
Gender		
Male	168 (66.4)	280 (60.9)
Female	85 (33.6)	180 (39.1)
Median age, SD (years)	68 (8.0)	65 (10.7)***
Education		
Elementary to high or technical school	111 (43.9)	227 (49.3)
Any college	80 (31.6)	144 (31.1)
Graduate or professional school	61 (24.1)	89 (19.3)
Smoking status^b^		
Never	79 (31.2)	146 (31.7)
Former	133 (52.6)	230 (50.0)
Current	40 (15.8)	84 (18.3)
Skin color		
Light	213 (84.2)	279 (60.6)***
Medium	38 (15.0)	180 (39.1)
Skin sun sensitivity^c^		
Severe sunburn with blistering	22 (8.7)	28 (6.1)***
Painful sunburn and then peeling	93 (36.8)	116 (25.2)
Mild sunburn with some tanning	111 (43.9)	234 (50.9)
Tan without sunburn	25 (9.9)	80 (17.4)
No. of lifetime painful sunburns^d^		
0	66 (26.1)	147 (32.0)***
1–2	49 (19.4)	134 (29.1)
3+	136 (53.8)	174 (37.8)
Prior keratinocyte cancer^e^		
Yes	74 (29.2)	37 (8.0)***
No	179 (70.8)	423 (91.9)
Glucocorticoid use^f^		
Yes	33 (13.0)	39 (8.5)
No	211 (83.4)	415 (90.2)
Transplant recipient		
Yes	6 (2.4)	1 (0.2)**
No	246 (97.2)	458 (88.6)

*P < 0.05, **P < 0.01, ***P < 0.001. *P* values obtained from X^2^, Fisher's exact, or Wilcoxon rank sum test (as appropriate) comparing sociodemographic and skin cancer risk factors between SCC cases and controls.

a Numbers may not sum to the overall total due to missing data. They were excluded from complete case analyses.

b Cigarette smoking status at 1 year prior to the reference or diagnosis date

c Sun sensitivity was defined as the skin reaction to 1 h of sun exposure the first time in the summer.

d Sunburns that caused pain for 2 or more days

e Prior keratinocyte cancer was defined as having had a previous squamous cell, basal cell, or both squamous cell and basal cell carcinoma.

f Glucocorticoid use was defined as having used oral steroid or corticosteroid medications (e.g., cortisone or prednisone) for 1 month or longer.

### Polyomaviruses and cutaneous squamous cell carcinoma

**Figure 1:**
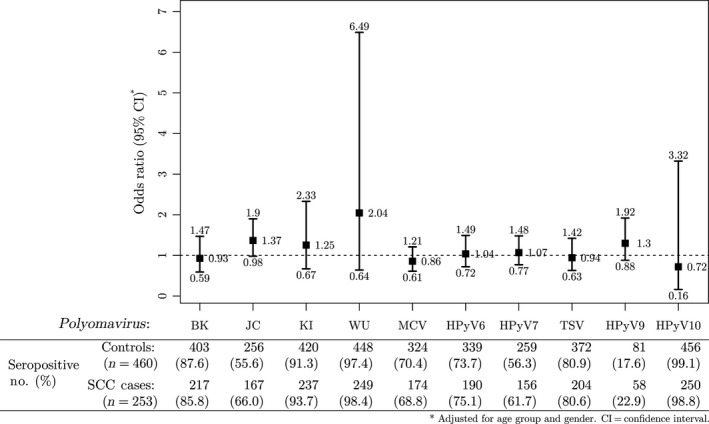
Odds ratios (95% confidence intervals as whiskers) for cutaneous squamous cell carcinoma (SCC) by seropositivity for each polyomavirus (PyV) type among 713 study participants from the New Hampshire Skin Cancer Study. The overall seroprevalences (%) and number of participants who were seropositive for each PyV are shown below the plot area separately for cases and controls.

Seroprevalences ranged from 17.6% for HPyV9 to 99.1% for HPyV10 among controls, and from 22.9% for HPyV9 to 98.8% for HPyV10 among cases (Fig. 1). All study participants were seropositive for at least one type of PyV, but cases (mean = 7.52, standard deviation = 1.40) were seropositive for a slightly greater number of PyVs than controls (mean = 7.30, standard deviation = 1.45; Wilcoxon rank sum test *P *= 0.049). However, we found no evidence of an increasing trend in SCC odds with increasing number of PyV types for which an individual tested seropositive, either using a categorical variable (*P* for trend = 0.42) or on a continuous scale (*P *= 0.32) (Table 2). Further, no association with SCC was observed for combined seropositivity to the known cutaneous PyVs (i.e., MCV, HPyV6, HPyV7, and TSV; Table S2).

**Table 2:** Odds ratios (95% confidence intervals) for cutaneous squamous cell carcinoma (SCC) by number of polyomavirus (PyV) types seropositive among 713 study participants from the New Hampshire Skin Cancer Study

No. of PyV types seropositive	Controls (*n *= 460), No. (%)	SCC cases (*n *= 253)	
		No. (%)	OR (95% CI)^a^
1–6	129 (28.0)	60 (23.7)	1.00 (referent)
7–8	236 (51.3)	128 (50.6)	1.04 (0.71–1.53)
9–10	95 (20.6)	65 (25.7)	1.21 (0.76–1.91)
*P* for trend			0.42
Continuous[mean No. (SD)]	7.30 (1.45)	7.52 (1.40)	1.06 (0.95–1.18)
*P*			0.32

OR, odds ratio; CI, confidence interval; SD, standard deviation.

a Adjusted for age group and gender.

In an analysis of SCC associations with antibodies to specific capsid antigens from each PyV type (classified as a dichotomous variable), an elevated odds of SCC was observed with seropositivity to JC of borderline statistical significance (OR = 1.37, 95% CI: 0.98–1.90; Fig. 1), and a positive trend in SCC risk was associated with increasing MFI quartiles of JC seroreactivity (*P* for trend = 0.04) (Table 3). A weakly positive association with SCC risk was observed for seropositivity to WU (OR = 2.04, 95% CI: 0.64–6.49) (Fig. 1), and a slightly inverse association with SCC risk was observed for seropositivity to HPyV10 (OR = 0.72, 95% CI: 0.16–3.32), but neither estimates achieved statistical significance. There were no trends in SCC risk by MFI quartiles for any PyV type but JC (Table S1).

**Table 3:** Odds ratios (95% confidence intervals) for cutaneous squamous cell carcinoma (SCC) by quartiles of JC polyomavirus (PyV) seroreactivity among 713 study participants from the New Hampshire Skin Cancer Study

PyV seroreactivity (MFI units)	Controls (*n *= 460), No. (%)	SCC cases (*n *= 253)	
		No. (%)	OR (95% CI)^a^
JC			
Quartile 1	115 (25.0)	51 (20.2)	1.00 (referent)
Quartile 2	115 (25.0)	43 (17.0)	0.79 (0.48–1.30)
Quartile 3	115 (25.0)	74 (29.2)	1.27 (0.80–1.99)
Quartile 4	115 (25.0)	85 (33.6)	1.41 (0.90–2.20)
*P* for trend			0.04

OR, odds ratio; CI, confidence interval.

a Adjusted for age group and gender.

SCC was more strongly related with seropositivity to JC among those reporting oral glucocorticoid use for 1 month or longer (OR = 1.88, 95% CI: 0.61–5.76) than those without a history of prolonged glucocorticoid use (OR = 1.37, 95% CI: 0.96–1.96)—albeit with limited statistical power (*P* for interaction = 0.50; Fig. S4). When excluding glucocorticoid users, the positive association between SCC risk and HPyV9 seropositivity was strengthened (OR = 1.49, 95% CI: 0.99–2.24). Interestingly, a weakly positive association with SCC risk was found for HPyV10 seropositivity among those seropositive for <2 *β* HPV types (OR = 1.45, 95% CI: 0.15–14.37), although with wide confidence intervals. We did not find any notable differences for the other PyVs according to glucocorticoid use, anatomical site of the SCC tumor, or seropositivity to *β* HPVs, in stratified analyses.

## Discussion

We measured antibodies against the first 10 discovered human PyVs in a large, population‐based case–control study to investigate the relation between multiple PyVs and SCC incidence. No clear associations were observed for most PyV types and SCC risk. However, we observed an intriguing elevated odds ratio for SCC associated with JC seropositivity that approached statistical significance, and a trend in risk by quartiles of JC seroreactivity. While no clear associations were detected, we had limited statistical power for many of these analyses due to the high PyV seroprevalence.

Several epidemiologic studies have raised the possibility of an oncogenic role for PyV infection in SCC development. Among 173 SCC cases and 300 controls in a clinic‐based case–control study conducted in Florida, USA, MCV seropositivity was related to an increased risk for SCC (OR = 1.58, 95% CI: 0.96–2.60), and the association was strongest among those with tumors containing MCV DNA (OR = 2.49, 95% CI: 1.03–6.04) 44. We did not observe evidence of an association with MCV seropositivity. An association of a similar magnitude with JC seropositivity was also observed in the Florida study (OR = 1.40, 95% CI: 0.89–2.20). However, unlike our study, there was no trend by quartiles of JC seroreactivity (*P* for trend = 0.44) 44.

In our case–control study design, we are unable to address the issue of ‘reverse causality’ common to studies aiming to elucidate the etiology of KC at or following diagnosis. Namely, it is possible that antibodies measured at the time of SCC diagnosis do not accurately reflect antibodies that would have been circulating in the early stages of carcinogenesis. Indeed, a large, prospective, Swedish study which drew upon samples donated to a biobank and cancer registry data found no association between SCC (OR = 1.0, 95% CI: 0.8–1.4) or BCC (OR = 0.9, 95% CI: 0.8–1.1) and prediagnostic JC seropositivity, with blood samples collected at least 1 month prior to diagnosis 46. However, the primary hypothesis of the Swedish study was an association between HPV and SCC or BCC, and thus used a multiplex assay optimized for the detection of antibodies against various HPV types, with JC included as a specificity control 46. A discrepancy in results may be attributed to both differing populations and assay optimization for human PyV types. Thus, further prospective data that include repeated measurements over time from each participant are needed.

Increased incidence of cancers in immunosuppressed patients, such as skin cancers 27, suggests a possible viral etiology that may be attributed to impaired immune surveillance of infections with oncogenic potential. Moreover, immune suppression could lead to viral reactivation and higher levels of antibodies, thus confounding any observed relationship between PyVs and SCC. A greater than expected proportion of transplant recipients are seropositive for KI and WU 69, 70, and MCV sequences are also more frequently found in the skin tumors of immunocompromised patients than those from immunocompetent individuals 71. TSV seroprevalence is likewise higher in renal transplant patients when compared to healthy participants 72, and has been causally linked to a rare disease of abnormal maturation of the hair follicles characterized by spiny lesions on the skin 73, 74. HPyV9 was first isolated from a kidney transplant recipient 53 and has been found at higher seroprevalence in immunocompromised patients 75. HPyV10 was isolated from a patient with warts, hypogammaglobulinemia, infections, and myelokathexis (WHIM) syndrome 54, an inherited immune deficiency with increased susceptibility to HPV‐induced warts and cancers 76, 77. BK and JC are known to reactivate under conditions of immunosuppression 78, 79. Indeed, JC seropositivity has been used as an indicator of immune status in prior studies 44. In a small, nested case–control study among transplant recipients, no excess risks for SCC following organ transplantation were found with antibody levels against PyVs (specifically, JC, KI, WU, MCV, HPyV6, and HPyV7) in serum drawn immediately prior to surgery (*n* = 149 SCC cases and 290 controls) 45. We observed increased odds of SCC associated with increasing JC antibody levels, which may reflect the detection of JC reactivation arising from immune dysregulation associated with skin cancer. Therefore, our findings of an association between JC serostatus and SCC could represent the predisposing role of immunodeficiency, rather than the virus itself. With limited statistical power, there was weak evidence that the association we found between SCC and JC seropositivity may be slightly stronger among those with prolonged oral glucocorticoid use, raising the possibility of effect modification rather than confounding by immunosuppression. However, further studies are needed.

MCV, TSV, HPyV6, and HPyV7 show evidence of skin tropism 36, yet we found no association with SCC risk. We could not assay for the presence of PyV DNA within SCC tumor tissues, which is a more direct indication of a potential association between PyVs and SCC. In the Florida study, MCV TAg DNA was isolated from ~40% of SCC tumors from patients, but they did not find BK, JC, KI, or WU TAg DNA sequences within SCC tumors 44. Other small studies have failed to detect DNA from JC in skin swabs 80 or skin biopsies from normal skin 81, common warts, Bowen's disease 82, BCC 83, SCC 81, 83, melanoma 81, 84, or cutaneous B‐cell lymphomas 85. However, JC TAg sequences were detected in Kaposi's sarcoma skin lesions, and has been amplified from 16% of healthy skin tissues 86, suggesting that these viruses can inhabit the skin even without being skin tropic 36 or the skin acting as a suitable host for viral propagation 87. A potential etiologic role of JC in the absence of JC DNA within tumors remains unexplained and warrants additional study.

All human PyVs discovered thus far encode proteins that allow them to act as oncoviruses. Indeed, the virus’ name is derived from the Greek *poly* meaning ‘many’ and *oma* referring to their induction of tumors in murine models 88. Truncating mutations in the large TAg of integrated MCV are characteristic of MCC tumors, with domains required for Rb‐induced cell transformation preserved, while those for viral replication and p53 binding 43 are eliminated resulting in no interaction with p53 89, thereby enhancing the likelihood of cell survival. Truncating mutations of the TAg have not been described in other human PyVs. Consequent to its transforming potential, the large TAg has been considered the main oncoprotein in PyVs 36. The small TAg and agnoprotein expressed by some viruses also exhibit oncogenic properties 36. The late regions of BK and JC encode an agnoprotein which exerts a tumorigenic effect through cell cycle dysregulation, interference with DNA repair processes, and increased chromosome instability 90, 91. Similar to SV40 92, BK, JC, and MCV encode a miRNA that downregulates large TAg expression, which may allow the virus to escape the immune system 93. Thus, there is some evidence for a putative mechanistic role for PyVs in human carcinogenesis, although further research is needed.

It has been theorized that PyVs may serve as cofactors for oncoviruses by acting on common tumorigenic targets, perturbing the cell cycle, assisting with immune evasion, or transactivating the promoters of co‐infecting viruses—although evidence of such an interaction is lacking 29. Interactions between PyVs and other viruses have been documented, such as the in vitro cooperation between JC and the HIV‐1 regulatory protein, Tat, that enhances JC transcription in glial cells 94, 95. Additionally, interactive effects between a murine PyV strain with Moloney murine leukemia virus have been shown to result in stunted growth only in co‐infected animals, possibly through a proinflammatory cytokine pathway 96. In our study, we were only able to assess concomitant seropositivity to *β* HPVs, and did not detect any consistent interactions with PyV types and SCC risk.

### Strengths and limitations

A strength of our study was the large number of histologically confirmed cases of incident invasive SCC identified through active population‐based surveillance, along with controls derived from the general US population. This type of study design decreases the opportunity for selection bias, and is more generalizable than clinic‐ or hospital‐based case–control studies. Still, the possibility of selection bias and residual confounding cannot be excluded, and the generalizability to non‐white populations is limited due to the study's location in an almost exclusively white US population. The use of multiplex serology to comprehensively measure a wide range of human PyVs was an additional study strength. The GST capture of recombinantly expressed VP1 capsid proteins has been found to be a reliable technique to assess PyV seroreactivity and has been used as a marker of PyV infection in prior studies 44, 58, 62. Our blood samples were collected following the skin cancer diagnosis, and thus PyV infection may have occurred during or following tumor development, and the influence of disease state on susceptibility to PyV infection and immune response cannot be disentangled from these data. We are unable to assess temporality or reverse causality, so the direction of any observed associations cannot be determined. PyV seropositivity does not necessarily correspond to the presence of PyV DNA in tumor tissues and seroreactivity is an indirect measure of infection. However, MCV viral load and antibody titer have been shown to have a strong positive monotonic correlation 97, with higher MFI values corresponding to MCV DNA‐positive SCC tumor tissues 44. Lastly, given the high seroprevalence of PyVs in the study population, power to detect differences in SCC risk for some PyVs was limited.

## Conclusions

In this population‐based case–control study, we examined the association between the first 10 discovered human PyVs and incidence of SCC using multiplex serology. Our findings, though limited, provide some support for the possibility that specific PyV types may play a role in the occurrence of SCC in the US population, but not PyV seropositivity in general.

## Conflict of Interest

None declared.

## Supporting information


**Table S1.** Odds ratios (95% confidence intervals) for cutaneous squamous cell carcinoma (SCC) by quartiles of polyomavirus (PyV) seroreactivity among 713 study participants from the New Hampshire Skin Cancer Study.

**Table S2.** Odds ratios (95% confidence intervals) for cutaneous squamous cell carcinoma (SCC) by seropositivity for all cutaneous polyomaviruses (PyV), and by number of cutaneous PyV types seropositive, among 713 study participants from the New Hampshire Skin Cancer Study.

**Figure S1.** Spearman rank correlation coefficients, ρ, between the median fluorescence intensity (MFI) values against each human polyomavirus (PyV) VP1 or T antigen (TAg) among 460 controls from the New Hampshire Skin Cancer Study, where *P<0.05, **P<0.01, ***P<0.001. Not all PyV TAgs were assayed, and MCV large TAg was assayed using the entire protein as well as with two fragments (exon 1 and exon 2) of the full length large TAg. The red triangle emphasizes the strong correlations between PyV TAgs.

**Figure S2.** Robustness of odds ratio (OR) estimates for cutaneous squamous cell carcinoma (SCC) by seropositivity for each polyomavirus (PyV) type among 713 study participants from the New Hampshire Skin Cancer Study, following adjustment for age group and gender. The cut points were varied from 50 to 450 median fluorescence intensity (MFI) units (x axis), and the resulting ORs were calculated using the new cutoffs (y axis). The red dots show the ORs using the recommended cutoff of 250 MFI units. The blue dots denote the cut points calculated using a frequency distribution analysis described in van der Meijden et al, 2013 (67). OR estimates for HPyV10 could not be accurately computed due to the viruses' high seroprevalence. The gray bands are the 95% confidence intervals (CI) about each OR.

**Figure S3.** Plot of odds ratios (95% confidence intervals as whiskers) for cutaneous squamous cell carcinoma (SCC) by seropositivity for each polyomavirus (PyV) type among study participants from the New Hampshire Skin Cancer Study, when excluding participants with a history of organ transplantation (“organ”, *n *= 1 control and 6 cases), restricting to participants with no previous skin cancers (“NMSC”, *n *= 423 controls and 179 cases), and excluding participants with a concomitant basal cell carcinoma (“BCC”, *n *= 22 cases), following adjustment for age group and gender. “Main” refers to unstratified risk estimates presented in Figure 1. The dashed line represents an OR=1.

**Figure S4.** Plot of odds ratios (95% confidence intervals as whiskers) for cutaneous squamous cell carcinoma (SCC) by seropositivity for each polyomavirus (PyV) type among study participants from the New Hampshire Skin Cancer Study, when stratified by oral glucocorticoid use for 1 month or longer (“yes” refers to use (*n* = 39 controls and 33 cases) and “no” to nonuse (*n* = 415 controls and 211 cases); as people with a history of glucocorticoid use may also have undergone organ transplantation, we restricted the analysis to those who were not organ transplant recipients), β HPV seropositivity (“≥2” (*n* = 125 controls and 82 cases) and “<2” (*n* = 335 controls and 171 cases) refers to number of β HPV seropositive), and SCC location (“head” (*n* = 146 cases) refers to SCC located on the head or neck, and “other” (*n* = 97 cases) refers to SCC located on other body parts), following adjustment for age group and gender. “Main” refers to unstratified risk estimates presented in Figure 1. OR and 95% CI were not computed for strata in which all participants were seropositive for the PyV of interest (represented by a solid vertical black line). The dashed line represents an OR=1.
