# Supplementary material for: Human polyomaviruses and incidence of cutaneous squamous cell carcinoma in the New Hampshire skin cancer study
Source: Cancer Med. 2016 Feb 21;5(6):1239–50. doi: 10.1002/cam4.674 (PMC4924382; doi:10.1002/cam4.674)
Supplement: Supplementary file 1 — Table S1. Odds ratios (95% confidence intervals) for cutaneous squamous cell carcinoma (SCC) by quartiles of polyomavirus (PyV) seroreactivity among 713 study participants from the New Hampshire Skin Cancer Study. [file CAM4-5-1239-s001.docx]

**Supplemental Table 1.** Odds ratios (95% confidence intervals) for cutaneous squamous cell carcinoma (SCC) by quartiles of polyomavirus (PyV) seroreactivity among 713 study participants from the New Hampshire Skin Cancer Study.

| **PyV seroreactivity**  **(MFI units)** | **Controls (n=460),**  **No. (%)** | **SCC Cases (n=253)** | |
| --- | --- | --- | --- |
|  |  | **No. (%)** | **OR (95% CI)*** |
| **BK** |  |  |  |
| Quartile 1 | 115 (25.0) | 64 (25.3) | 1.00 (referent) |
| Quartile 2 | 115 (25.0) | 64 (25.3) | 1.01 (0.65-1.57) |
| Quartile 3 | 115 (25.0) | 63 (24.9) | 0.99 (0.64-1.55) |
| Quartile 4 | 115 (25.0) | 62 (24.5) | 1.15 (0.74-1.81) |
| *P* for trend |  |  | 0.58 |
| **KI** |  |  |  |
| Quartile 1 | 115 (25.0) | 52 (20.6) | 1.00 (referent) |
| Quartile 2 | 115 (25.0) | 83 (32.8) | 1.62 (1.04-2.53) |
| Quartile 3 | 115 (25.0) | 61 (24.1) | 1.27 (0.80-2.01) |
| Quartile 4 | 115 (25.0) | 57 (22.5) | 1.08 (0.67-1.72) |
| *P* for trend |  |  | 0.89 |
| **WU** |  |  |  |
| Quartile 1 | 115 (25.0) | 74 (29.2) | 1.00 (referent) |
| Quartile 2 | 115 (25.0) | 49 (19.4) | 0.68 (0.43-1.07) |
| Quartile 3 | 115 (25.0) | 68 (26.9) | 0.95 (0.62-1.47) |
| Quartile 4 | 115 (25.0) | 62 (24.5) | 0.85 (0.55-1.32) |
| *P* for trend |  |  | 0.78 |
| **MCV** |  |  |  |
| Quartile 1 | 115 (25.0) | 66 (26.1) | 1.00 (referent) |
| Quartile 2 | 115 (25.0) | 77 (30.4) | 1.08 (0.70-1.66) |
| Quartile 3 | 115 (25.0) | 41 (16.2) | 0.57 (0.35-0.93) |
| Quartile 4 | 115 (25.0) | 69 (27.3) | 0.97 (0.63-1.51) |
| *P* for trend |  |  | 0.37 |
| **HPyV6** |  |  |  |
| Quartile 1 | 115 (25.0) | 54 (21.3) | 1.00 (referent) |
| Quartile 2 | 115 (25.0) | 64 (25.3) | 1.13 (0.72-1.79) |
| Quartile 3 | 115 (25.0) | 65 (25.7) | 1.20 (0.76-1.89) |
| Quartile 4 | 115 (25.0) | 70 (27.7) | 1.22 (0.78-1.92) |
| *P* for trend |  |  | 0.37 |
| **HPyV7** |  |  |  |
| Quartile 1 | 115 (25.0) | 51 (20.2) | 1.00 (referent) |
| Quartile 2 | 115 (25.0) | 62 (24.5) | 1.18 (0.74-1.88) |
| Quartile 3 | 115 (25.0) | 57 (22.5) | 0.90 (0.56-1.44) |
| Quartile 4 | 115 (25.0) | 83 (32.8) | 1.41 (0.90-2.21) |
| *P* for trend |  |  | 0.25 |
| **TSV** |  |  |  |
| Quartile 1 | 115 (25.0) | 60 (23.7) | 1.00 (referent) |
| Quartile 2 | 115 (25.0) | 66 (26.1) | 0.98 (0.62-1.54) |
| Quartile 3 | 115 (25.0) | 70 (27.7) | 1.09 (0.70-1.72) |
| Quartile 4 | 115 (25.0) | 57 (22.5) | 1.02 (0.64-1.62) |
| *P* for trend |  |  | 0.82 |
| **HPyV9** |  |  |  |
| Quartile 1^a^ | 114 (24.8) | 53 (20.9) | 1.00 (referent) |
| Quartile 2 | 113 (24.6) | 72 (28.4) | 1.31 (0.83-2.05) |
| Quartile 3 | 118 (25.6) | 48 (19.0) | 0.85 (0.52-1.37) |
| Quartile 4 | 115 (25.0) | 80 (31.6) | 1.41 (0.90-2.21) |
| *P* for trend |  |  | 0.35 |
| **HPyV10** |  |  |  |
| Quartile 1 | 115 (25.0) | 66 (26.1) | 1.00 (referent) |
| Quartile 2 | 115 (25.0) | 72 (28.4) | 1.14 (0.74-1.76) |
| Quartile 3 | 115 (25.0) | 50 (19.8) | 0.82 (0.52-1.31) |
| Quartile 4 | 115 (25.0) | 65 (25.7) | 1.16 (0.75-1.82) |
| *P* for trend |  |  | 0.84 |

^*^ Adjusted for age group and gender. OR=odds ratio, CI=confidence interval.

^a^ Controls may not be evenly distributed within quartiles due to uneven data distribution.
