# Supplementary material for: Human polyomaviruses and incidence of cutaneous squamous cell carcinoma in the New Hampshire skin cancer study
Source: Cancer Med. 2016 Feb 21;5(6):1239–50. doi: 10.1002/cam4.674 (PMC4924382; doi:10.1002/cam4.674)
Supplement: Supplementary file 2 — Table S2. Odds ratios (95% confidence intervals) for cutaneous squamous cell carcinoma (SCC) by seropositivity for all cutaneous polyomaviruses (PyV), and by number of cutaneous PyV types seropositive, among 713 study participants from the New Hampshire Skin Cancer Study. [file CAM4-5-1239-s002.docx]

**Supplemental Table 2.** Odds ratios (95% confidence intervals) for cutaneous squamous cell carcinoma (SCC) by seropositivity for all cutaneous polyomaviruses (PyV), and by number of cutaneous PyV types seropositive, among 713 study participants from the New Hampshire Skin Cancer Study.

| **PyV seroreactivity** | **Controls (n=460),**  **No. (%)** | **SCC Cases (n=253)** | |
| --- | --- | --- | --- |
|  |  | **No. (%)** | **OR (95% CI)^*^** |
| **Cutaneous**^‡^ |  |  |  |
| Seronegative | 313 (68.0) | 168 (66.4) | 1.00 (referent) |
| Seropositive | 147 (32.0) | 85 (33.6) | 0.94 (0.67-1.32) |
|  |  |  |  |
| Continuous |  |  | 0.98 (0.85-1.14) |
| *P* |  |  | 0.83 |

^*^ Adjusted for age group and gender. OR=odds ratio, CI=confidence interval.

^‡^ Cutaneous PyVs consisted of MCV, HPyV6, HPyV7, and TSV. Those who were seropositive for all 4 cutaneous PyVs were compared to those seropositive for ≤3 cutaneous PyVs.
