# Supplementary figures and images for: Human polyomaviruses and incidence of cutaneous squamous cell carcinoma in the New Hampshire skin cancer study
Source: Cancer Med. 2016 Feb 21;5(6):1239–50. doi: 10.1002/cam4.674 (PMC4924382; doi:10.1002/cam4.674)

Supplemental Figure 1

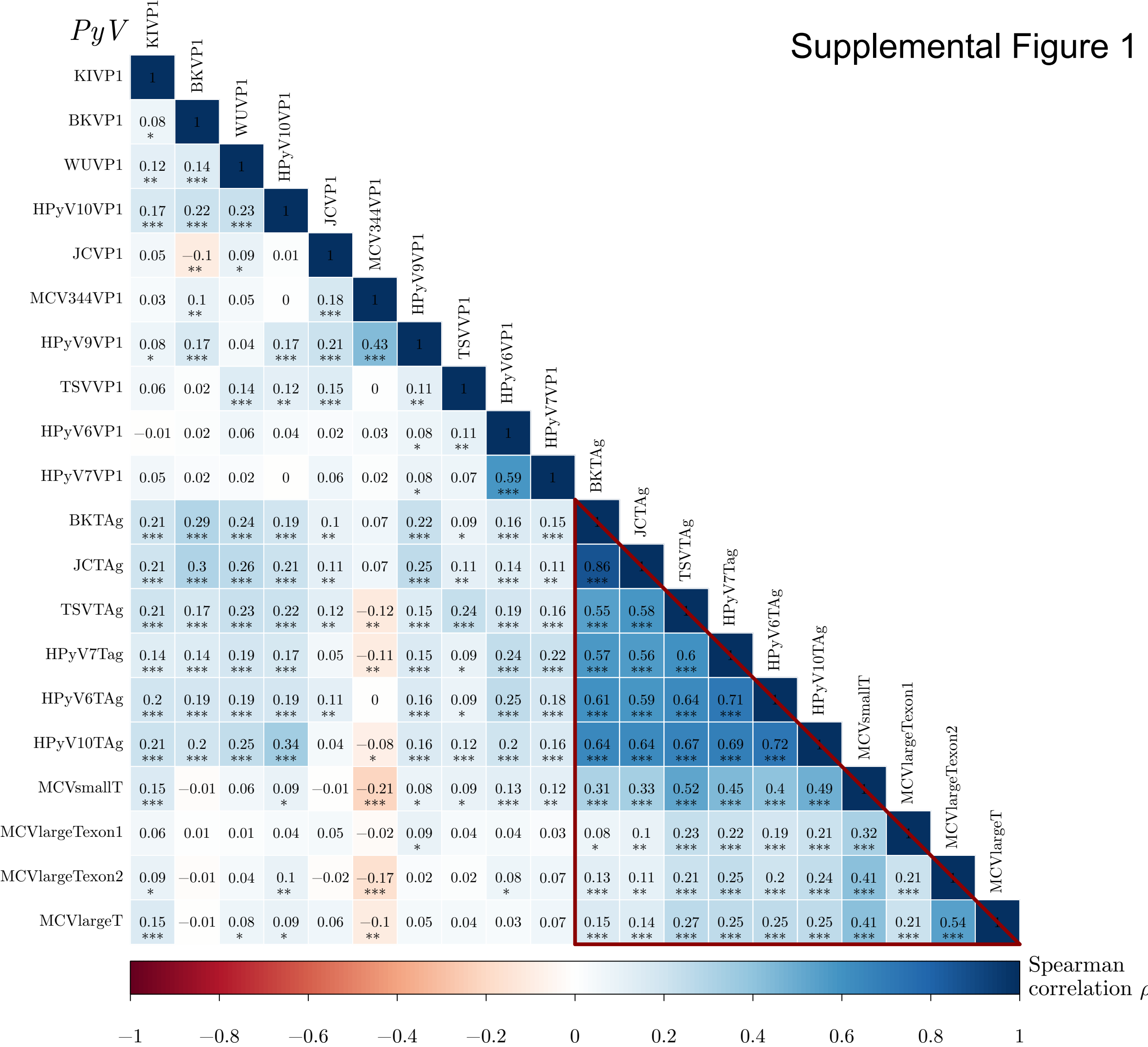

Supplement: Supplementary file 3 — Figure S1. Spearman rank correlation coefficients, ρ, between the median fluorescence intensity (MFI) values against each human polyomavirus (PyV) VP1 or T antigen (TAg) among 460 controls from the New Hampshire Skin Cancer Study, where *P<0.05, **P<0.01, ***P<0.001. Not all PyV TAgs were assayed, and MCV large TAg was assayed using the entire protein as well as with two fragments (exon 1 and exon 2) of the full length large TAg. The red triangle emphasizes the strong correlations between PyV TAgs. [file CAM4-5-1239-s003.pdf]

BK

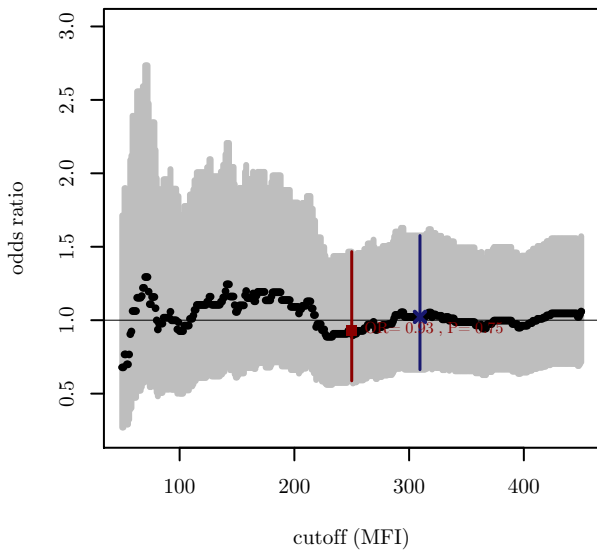

JC

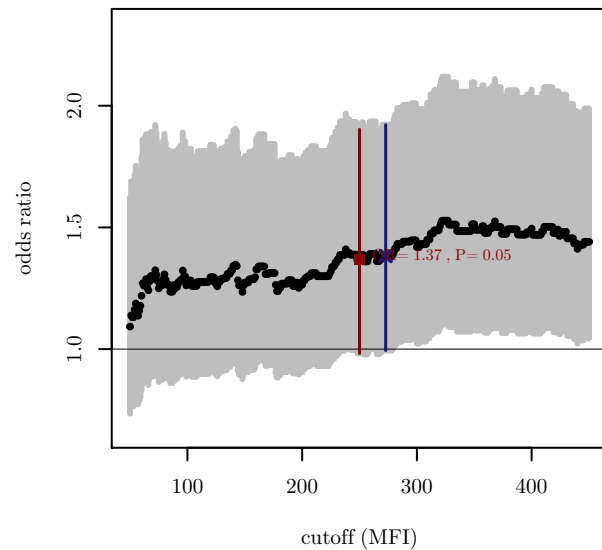

KI

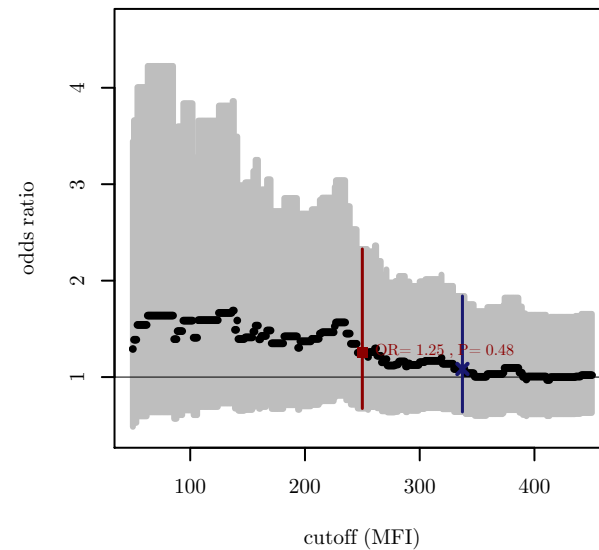

WU

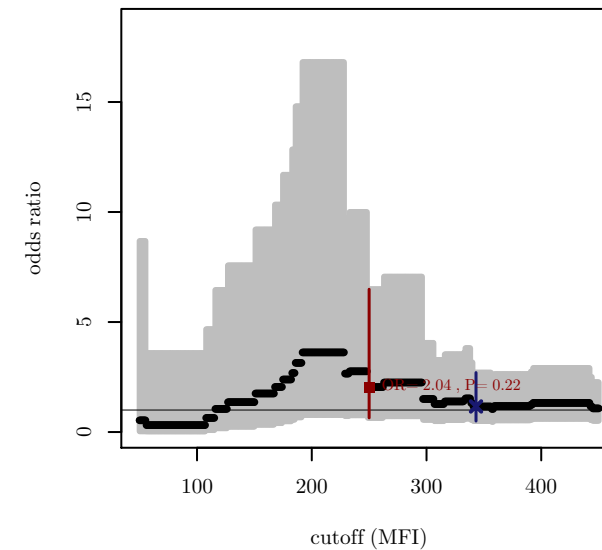

MCV

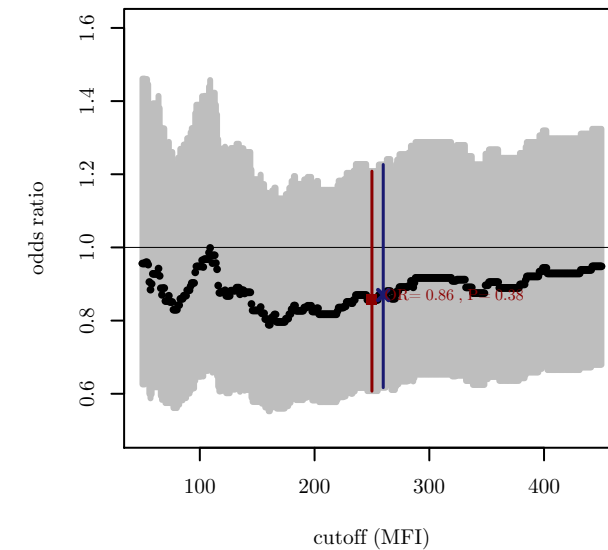

HPyV6

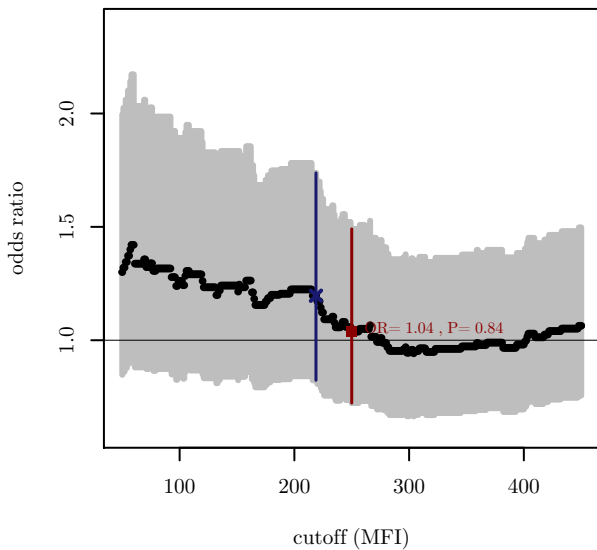

HPyV7

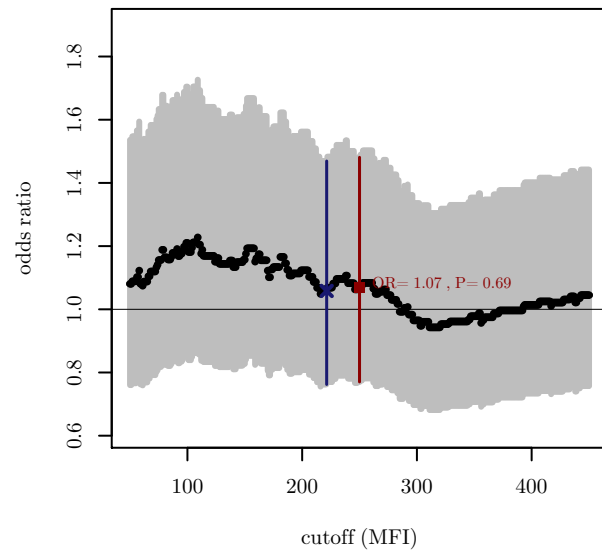

TSV

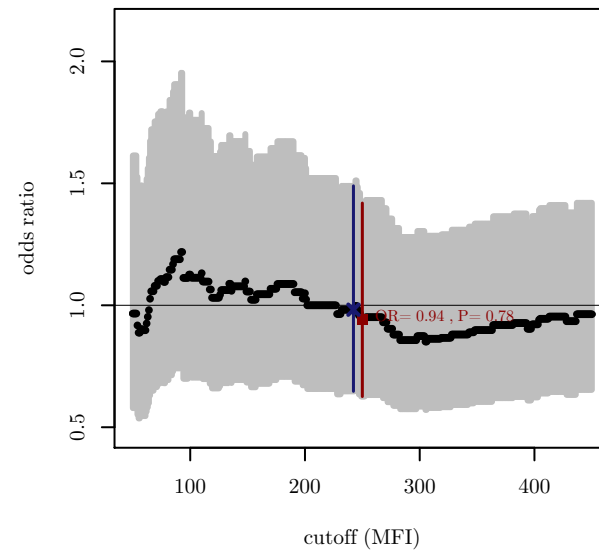

HPyV9

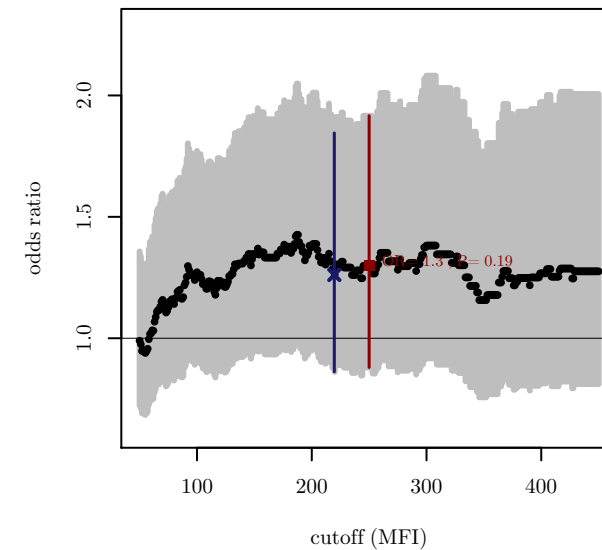Supplemental  
Figure 2

Supplement: Supplementary file 4 — Figure S2. Robustness of odds ratio (OR) estimates for cutaneous squamous cell carcinoma (SCC) by seropositivity for each polyomavirus (PyV) type among 713 study participants from the New Hampshire Skin Cancer Study, following adjustment for age group and gender. The cut points were varied from 50 to 450 median fluorescence intensity (MFI) units (x axis), and the resulting ORs were calculated using the new cutoffs (y axis). The red dots show the ORs using the recommended cutoff of 250 MFI units. The blue dots denote the cut points calculated using a frequency distribution analysis described in van der Meijden et al, 2013 (67). OR estimates for HPyV10 could not be accurately computed due to the viruses' high seroprevalence. The gray bands are the 95% confidence intervals (CI) about each OR. [file CAM4-5-1239-s004.pdf]

Supplemental Figure 3

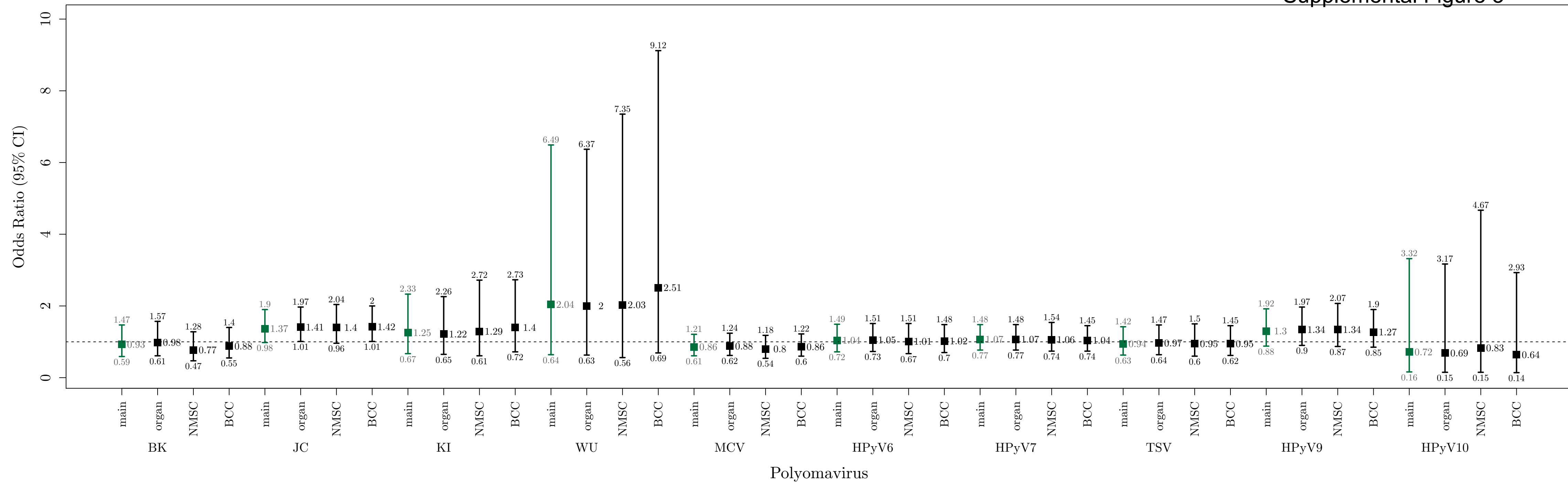

Supplement: Supplementary file 5 — Figure S3. Plot of odds ratios (95% confidence intervals as whiskers) for cutaneous squamous cell carcinoma (SCC) by seropositivity for each polyomavirus (PyV) type among study participants from the New Hampshire Skin Cancer Study, when excluding participants with a history of organ transplantation (“organ”, n = 1 control and 6 cases), restricting to participants with no previous skin cancers (“NMSC”, n = 423 controls and 179 cases), and excluding participants with a concomitant basal cell carcinoma (“BCC”, n = 22 cases), following adjustment for age group and gender. “Main” refers to unstratified risk estimates presented in Figure 1. The dashed line represents an OR=1. [file CAM4-5-1239-s005.pdf]

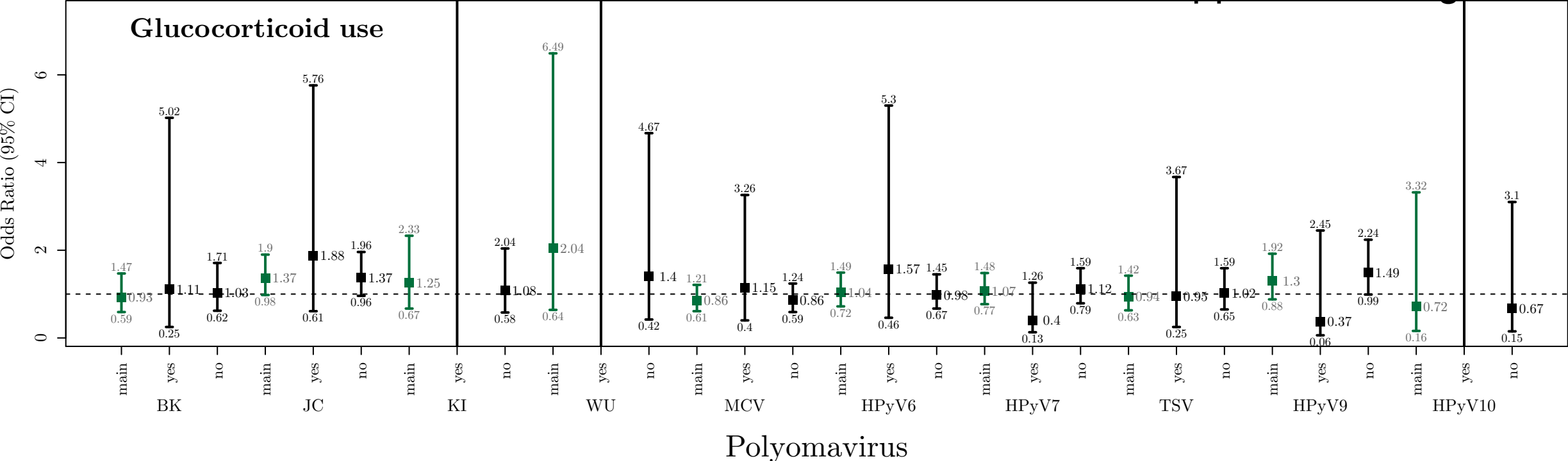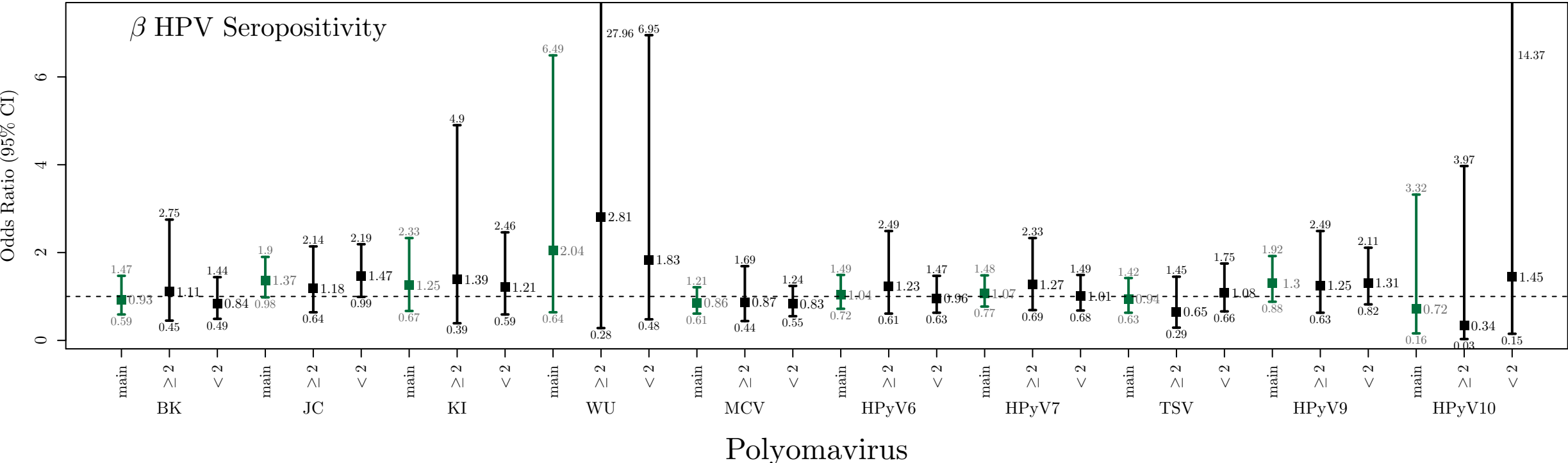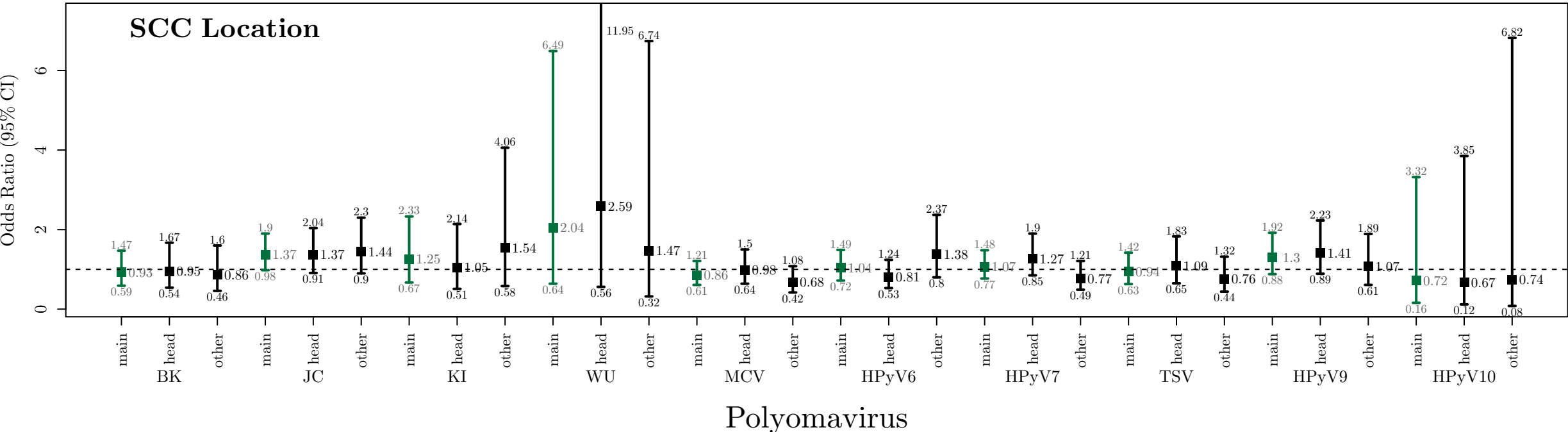

Supplement: Supplementary file 6 — Figure S4. Plot of odds ratios (95% confidence intervals as whiskers) for cutaneous squamous cell carcinoma (SCC) by seropositivity for each polyomavirus (PyV) type among study participants from the New Hampshire Skin Cancer Study, when stratified by oral glucocorticoid use for 1 month or longer (“yes” refers to use (n = 39 controls and 33 cases) and “no” to nonuse (n = 415 controls and 211 cases); as people with a history of glucocorticoid use may also have undergone organ transplantation, we restricted the analysis to those who were not organ transplant recipients), β HPV seropositivity (“≥2” (n = 125 controls and 82 cases) and “<2” (n = 335 controls and 171 cases) refers to number of β HPV seropositive), and SCC location (“head” (n = 146 cases) refers to SCC located on the head or neck, and “other” (n = 97 cases) refers to SCC located on other body parts), following adjustment for age group and gender. “Main” refers to unstratified risk estimates presented in Figure 1. OR and 95% CI were not computed for strata in which all participants were seropositive for the PyV of interest (represented by a solid vertical black line). The dashed line represents an OR=1. [file CAM4-5-1239-s006.pdf]
